# The effect of repeated torque tightening on total lengths of implant abutments in different internal implant‒abutment connections

**DOI:** 10.15171/joddd.2017.020

**Published:** 2017-06-21

**Authors:** Fariba Saleh Saber, Nader Abolfazli, Soheil Jannatii Ataei, Mahsa Taghizade Motlagh, Vahede Gharekhani

**Affiliations:** ^1^Dental and Periodontal Research Center, Tabriz University of Medical Sciences, Tabriz, Iran; ^2^Department of Prosthodontics, Faculty of Dentistry, Tabriz University of Medical Sciences, Tabriz, Iran; ^3^Department of Periodontics, Faculty of Dentistry, Tabriz University of Medical Sciences, Tabriz, Iran

**Keywords:** Axial displacement, morse hex taper, settling effect, tightening torque

## Abstract

***Background.*** Since the misfit of crown has an important role in clinical performance of implant-supported prostheses, and due to the impact of the settling effect on misfit, the aim of this study was to investigate the impact of torque forces on the total lengths of narrow and short implant abutments in different internal implant‒abutment connections.

***Methods.*** In four different implant‒abutment connections, 8 analog implants with a normal diameter (4 mm) and narrow abutment (4.5 mm) were selected from groups of internal hex, internal octagon, morse hex 6° and morse hex 11°. Each of them was mounted within plaster type IV, and 32 samples were obtained. Then, the amount of vertical displacement was measured by closing the impression copings and applying torques of 20 25 and 30 Ncm. This stage was repeated for the abutment. In the next stage, the resin pattern was built and measurements were performed after applying the torques mentioned. Finally, after making the frame, this stage was repeated, and the settling effect was statistically analyzed with ANOVA.

***Results.*** In the stages of impression coping, resin pattern and final prosthesis, HEXAGONE had significantly the highest and OCTAGONE had the lowest rates of settling, and the settling of morse hex 11° and 6° was between them.

***Conclusion.*** Octagon implant had significantly the lowest settling in various clinical and laboratory stages by applying different torques.

## Introduction

The implant‒abutment connection is very important since misfit is one of the possible reasons cited for biological and mechanical effects.^1^ On the other hand, the construction of implant components and the impact of clinical and laboratory stages create misfit between the implant and prosthesis. A misfit between implant and abutment will create tensile and compressive forces that will be exerted on the restoration, leading to prosthesis and abutment screw loosening, restoration fracture, bone microfractures around the implant and even the fracture of the implant body.^2,3^In addition, misfit is a suitable place for aggregation of microorganisms, leading to inflammation of soft tissues around the implant. Therefore, the connection between the suprastructure and implant platform or abutment is an important factor in the success of implant-supported prostheses. Even a small amount of misfit can cause changes in the geometry of the screw and the strain exerted on the screw. Major risks associated with crestal bone loss are prosthetic screw loosening and implant fracture.^2,4^

The implant‒abutment connection is basically classified into two general groups of internal and external. Branemark external implant‒abutment connection layout was widely used in the past. Problems such as screw loosening and rotational misfit at the implant‒abutment contact, led to the emergence of implants with internal connections.^2,5^

Of implants with internal connections, we can mention morse hex taper, internal octagon and internal hexagon; the internal hexagonal implants are the most common type available with a hexagon shape at the implant‒abutment connection point. Morse taper implants include a tapered body in the abutment that is placed inside the hollow of the tapered implant. Implant‒abutment connection point in internal octagonal implants is an octagon shape.^4,5^

When the abutment is placed in the implant, settling effect occurs with varying degrees by applying different torques to all the systems. It increases depending on the micro-roughness between metal surfaces of implant‒abutment connection.^6^ The mechanism of settling effect is based on the fact that there is no completely smooth surface, and settling happens to smoothen the rough points under pressure because when the primary forces are used, these points are the only surfaces in contact. Abrasion of the contact surfaces makes the two surfaces closer.^7^ This vertical displacement in the axial axis occurs in all the clinical and laboratory stages of implant-supported prostheses. Thus, the axial position of the abutment in the oral cavity and the master cast is different. Application of different torques during the work process and the emergence of discrepancy in the position of abutment result in the loss of passive fit in the suprastructure, ultimately leading to implant‒prosthesis misfit.^8^

Branemark stated the acceptable amount of misfit is 10 microns, but according to Jemt et al, misfit in the external connection, is clinically acceptable up to 150 microns. There is no report on the misfit clinical tolerance level for the internal connection.^4,6^

Narrow implants are structurally weaker than the implants with normal diameter. A 20% reduction in the diameter of implants leads to a 50% reduction in their resistance against failure. These implants have limitations such as less surface area, decreased fatigue strength and a high risk of screw loosening. However, in cases like periodontal disease and trauma, as well as in the anterior areas, use of these narrow implants is inevitable.^9^ Providing passive fit is inevitable in the screw prosthesis because there is no compensation for misfit.^10^ For example, clinical procedures such as impression, settling effect of the impression copings and exertion of force can lead to inaccuracies in impressions in implants with internal connection. In the laboratory and the oral cavity, impression coping will be connected to the implant analog by a hand wrench. While, in the final connection of prosthesis, abutment will be connected to the implant by applying torque. Abutment-level impression is recommended in the implants with an internal connection in order to reduce the settling effect. However, in cases where interocclusal space is limited, implant-level impression of screw prosthesis is inevitable. In this case, access to passive fit will be more questionable.^11^

Since the crown misfit has an important role in the clinical performance of implant-supported prostheses, and the dentist has access to a variety of implants, and due to the impact of the settling effect on misfit, and availability of limited laboratory studies on settling effect, especially single prosthesis in morse hex taper implants, and narrow and short abutments, the aim of this study was to investigate the impact of torque forces on the length of the implant‒abutment set in different types of implant‒abutment connections.

## Methods

In this study, the implant analog and abutment of the Periosave system (Mana Idea Bartar, France) were used. The normal diameter of the implant analog (4 mm) and narrow abutment (4.5 mm) were selected for all the groups, and various types of internal implant‒abutment connection were tested.

The internal connections studied included internal octagon, internal hexagon, morse hex taper 11° and morse hex taper 6°. Eight implant analogs were selected from each group and placed in dental stone type IV (GC Fujirock EP; GC Europe, Leuven, Belgium). We used a surveyor (Saeyang, SDS 103, China) to ensure the uniformity of the vertical placement of analogs. Thus, eight master casts were obtained for each group.

**Figure 1:**
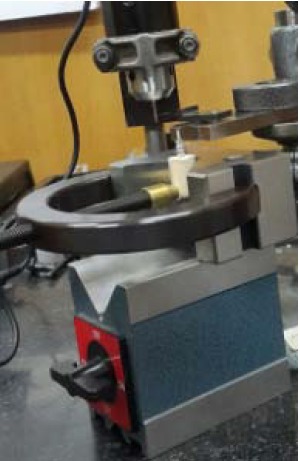
Application of torque.

**Figure 2:**
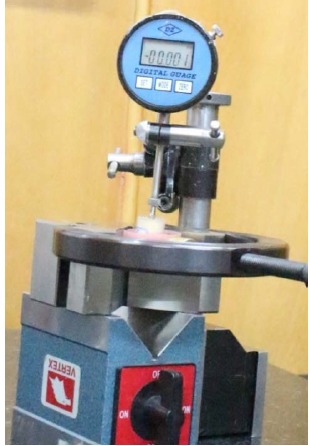
Axial displacement measurement

At this stage, an impression coping was connected to each implant analog; then each set was fixed with a clamp, and a manual wrench^11^ with a minimum force was used for impression coping connection. Then using a digital torque controller (Lutron TQ-8800. Taiwan), 20, 25 and 30 Ncm torques were applied (Figure 1). The axial displacement of each set of implant analogs and impression copings was measured by one operator using a digital indicator (Mitutoyo, Kawasaki, Japan) with 0.001-mm resolution (Figure 2).

In the next stage, the abutments were connected to the relevant implant analogs on the master cast. To simulate the clinical situation, since in most cases there is low crown height space in the maxillary arch, the abutment height was set to about 5 mm. Then, autoploymerized resin (Pattern resin; GC Corporation, Tokyo, Japan) was used for making a resin abutment in a way that the resin covered the abutment surface completely, and the resin layer thickness was about 2 mm. The resin was used layer by layer to reduce polymerization shrinkage. When the resin was polymerized, 20, 25 and 30 Ncm torques were applied using a digital torque controller; then the axial displacement of each sample was measured by one operator. Next, the resin was invested in‏ phosphate-bonded investment (Ticonium Albany, NY, USA), and casting was carried out by nickel‒chromium alloy (BEGO, Germany). Then the 20, 25 and 30 Ncm torques were applied.^12^ At this stage, the axial displacement of implant replica, uncemented final prosthesis assemblies, was measured.

All the measurements were repeated three times and a mean value was reported.

## Statistical analysis

Data were analyzed using descriptive statistical methods (mean ± SD) and repeated-measures ANOVA (MANOVA) and one-way ANOVA, followed by appropriate post hoc tests for significance, using SPSS 17. In this study, P<0.05 was considered statistically significant.

## Results

In the present study, the settling amount was investigated in different laboratory stages and it was shown that a certain amount of settling was used in different torques in all the implant cases.

**Figure 3:**
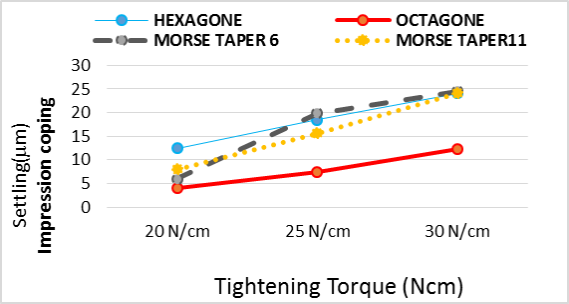
Comparison of settling in 4 types of implants under the effect of three different torques in impression coping connection.

In impression coping connection (Table 1, Figure 3):

**Table 1:** Comparison of settling in terms of the type of implant‒abutment connection and the application of torque in each of the connections

	Torque	**HEXAGONE**	**OCTAGONE**	**MORSE TAPER 6**	**MORSE TAPER11**	**p value**
**Impression coping **	20	12.5^a^± .53	4.12^d^ ± .99	6^c^± .92	8^b^± .75	<.001
	25	18.50^b^±.76	7.50^d^± 1.20	19.88^a^±1.55	15.63^c^ ±1.19	<.001
	30	24.13^a^ ±.64	12.25^b^±1.98	24.5^a^±.53	24.13^a^ ±1.64	<.001
**Resin abutment**	20	11.62 ^a^±.74	3.25^c^±.46	2.75^c^ ±.46	7.12^b^± .35	<.001
	25	23.25^a^ ±.88	7.75^d^±.46	13.25^c^ ±.46	17.12^b^ ±.35	<.001
	30	29.5^a^±.53	15.62^d^±.74	20.75^c^±.46	27.12^b^±.35	<.001
**frame**	20	11.5^a^±.75	3.5 ^c^±.53	3.25^c^ ±.46	7.12^b^±.35	<.001
	25	22.5^a^±.53	7.37^d^±.51	11.25^c^±.46	17.12^b^±.35	<.001
	30	30.25^a^ ±.70	14.5^d^ ±.75	20.5^c^ ±1.06	29^b^±0	<.001
**abutment**	5	0	0	0	0	
	10	9.00^a^±1.2	3.63^b^±2.5	4.63^b^±3.5	4.13^b^±4.6	<.001
	20	17.75^a^±1.04	8.38^c^±3.42	11.63^b^±0.92	15.25^a^±0.89	<.001
	25	23.63^a^±.74	12.75^c^±.17	18.50^b^±.76	21.63^a^±.92	<.001
	**30**_1_	31.5^a^±3.89	16.13^c^ ±5.82	22.50^b^± 0.53	24.50^b^ ±0.76	<.001
	**30**_2_	34.7^a^ ±3.77	18.13 ^c^±5.79	25.38^b^ ±0.74	24.50^b^± 0.76	<.001
	**30**_3_	34.7^a^ ±3.77	18.13 ^c^±5.79	25.38^b^ ±0.74	24.50^b^± 0.76	<.001
	**30**_4_	34.7^a^ ±3.77	18.13 ^c^±5.79	25.38^b^ ±0.74	24.50^b^± 0.76	<.001
	**30**_5_	34.7^a^ ±3.77	18.13 ^c^±5.79	25.38^b^ ±0.74	24.50^b^± 0.76	<.001
	P value*	<.001	<.001	<.001	<.001	

P-value obtained from one-way ANOVA P-value* obtained from repeated-measures ANOVA in order to compare the rate of settling in 5 times of use of torque 30 a, b, c, … Tukey test for grouping implants in terms of settling in every torque. Identical letters show a lack of significance.

In 20 Ncm torque: Hexagon and octagon implants had significantly the highest and lowest settling, respectively.

In 25 Ncm torque: Morse taper 6° and octagon implants had significantly the highest and lowest settling, respectively.

In 30 Ncm torque: Hexagon, morse taper 6˚ and morse taper 11˚ had the same settling which was significantly higher than octagon.

**Figure 4:**
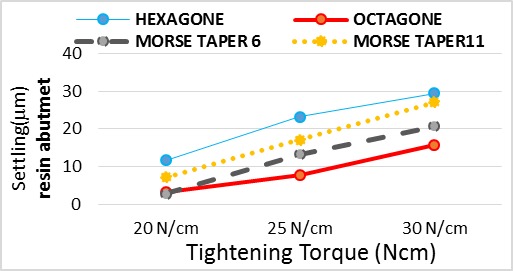
Comparison of settling in 4 types of implant‒abutment connection under the effect of three different torques in resin abutment.

In resin abutment (Table 1, Figure 4):

In 20 Ncm torque: Hexagon implant had significantly the highest settling while octagon and morse taper 6° implants had significantly the lowest settling.

In 25 Ncm torque: Hexagon and octagon implants had significantly the highest and lowest settling, respectively.

In 30 Ncm torque: Hexagon and octagon implants had significantly the highest and lowest settling, respectively.

**Figure 5:**
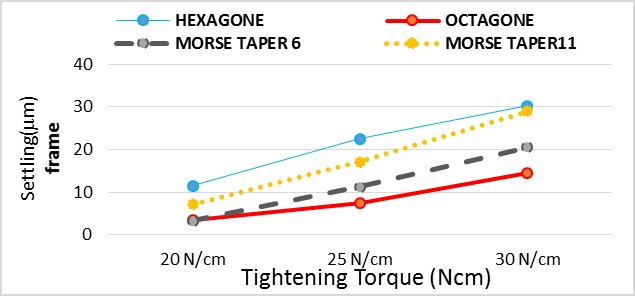
Comparison of settling in 4 types of implant‒abutment connection under the effect of three different torques in the final prosthesis.

In final prosthesis (frame) (Table 1, Figure 5):

In 20 Ncm torque: Hexagon implant had significantly the highest settling while octagon and morse taper 6° implants had significantly the lowest settling.

In 25 Ncm torque: Hexagon and octagon implants had significantly the highest and lowest settling, respectively.

In 30 Ncm torque: Hexagon and octagon implants had significantly the highest and lowest settling, respectively.

**Figure 6:**
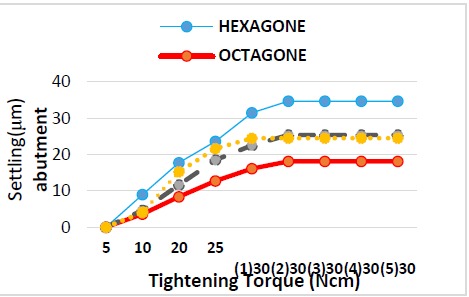
Comparison of settling in 4 types of implants under the effect of different torques in the abutment.

In abutment connection (Table1, Figure 6):

In 20 Ncm torque: Hexagon and morse taper 11° implants had significantly the highest settling while octagon and morse taper 6° implants had the identical settling.

In 25 Ncm torque: Hexagon and morse taper 11° implants had significantly the highest settling while octagon implant had significantly the lowest settling.

In 30_1_ Ncm torque: Hexagon and octagon implants had significantly the highest and lowest settling, respectively.

In 30_2_ Ncm torque: Hexagon and octagon implants had significantly the highest and lowest settling, respectively.

In 30 Ncm torque: There was a significant difference between the amount of settling in the first and second repetitions, but there were no significant differences in settling between torque repetitions of 2, 3, 4 and 5.

## Discussion

Settling occurs when the rough points on metal surfaces that are in contact with each other become smooth under the influence of forces because when the initial torque is applied, these points are the only surfaces in contact. In the implant prosthesis, the phenomenon of settling occurs between the fixture and abutment screw threads, between the fixture head and the lower part of the abutment, and between the abutment screw head and abutment.^6,13^In the present study, the settling amount was investigated in different laboratory stages and it a certain amount of settling was shown in different torques used in all the cases of implants. According to the results of this study, the lowest settling happened in octagonal implants.

In the multi-stage process, impression coping, resin abutment and final prosthesis, varying amounts of settling were shown.

Vertical displacement of impression coping in different connections used in our study was higher in Hex and morse hex 6° in 25 Ncm torque compared to morse taper 11° and octagon. However, in 30 Ncm torque, the final value of settling was identical in all the 3 types of connections of hex, morse hex 6° and 11°, and was higher compared with the octagon.

In this study, all the impression copings used were of two-piece type. But in studies by Kim et al and Lee et al both two-piece and single-piece copings were used. They concluded that vertical displacement was higher in two-piece types compared to single-piece ones, due to higher frictional resistance of components in the single-piece type. In addition, immersion of the available two-piece types together increased settling.^14,15^

In the present study, a certain amount of abutment settling occurred within the implant, in different torques, in all the cases of implants used. According to the results of this study, the lowest settling occurred in octagon.

The importance of settling differences between different types of implant connections is due to the passive prosthesis fit and avoiding loosening of screws. Clinicians usually use implant-level impression technique to fabricate long-span fixed prosthesis; therefore, they must consider the possibility of discrepancy in an abutment position due to settling. In the implant-level impression technique, at least four connections (fixtures‒impression coping, impression coping‒laboratory analog, laboratory analog‒abutment, abutment‒fixture) are required between the different components. In the present study, a difference in settling was observed between the torques 10 to 30 Ncm. The difference in the torques exerted in different stages results changes in vertical position of the abutment in the oral cavity compared to the master cast. These vertical discrepancies are associated with rotational freedom, this means that the horizontal and rotational discrepancies can cause strains in superstructures; if it does not cause bone loss, it will lead to mechanical complications, like screw, framework and the veneer fracture.^14^

Squier et al (2002) reported a decrease in loosening torque in abutments with a flat surface in implants with internal connection, but settling effect was not assessed in their study.^16^In this study, the process of manufacturing and torqueing was performed by one operator. When applying the torque forces, the amount of settling in hex abutments was more than morse hex, and it was more than octagon.

This suggests that increasing the angles of abutment‒implant connection will further reduce the settling effect. On the other hand, in the study by Lee et al axial displacement in two internal connections of morse 11° and external hex was examined. They concluded that vertical displacement in morse 11° was more than that in external hex. It is due to a lack of certain vertical stop in morse 11°, wherer the taper abutment enters the internal parts of the implant, leading to vertical displacement.^14^ However, based on our study, combination of morse hex exhibited lower vertical displacement compared with hex. It can be said in justification that vertical stop, which is identical in morse hex and hex and causes vertical displacement in morse hex, is not more than hex. Due to the addition of morse to hex and an overall increase in the surface that withstands the forces, a reduction in displacement was seen in morse hex compared to hex. In the comparison of morse hex 6° and morse hex 11° where the vertical displacement in morse hex 6° was less than morse hex 11°, it can be said, due to the decrease in taper, that the trough of the abutment‒implant is smaller.

Settling effect occurs in two stages: first during tightening of the screw and second stage during exerting occlusal loads. Therefore, any clinical and laboratory process that requires abutment screw tightening could lead to uncontrolled displacement.^11^ In all the cases, the amount of axial displacement in the resin abutment and final prosthesis (frame) was similar, different from the results of a study by Kim et al.^15^ In their study, the axial displacement of both external and internal connection groups in the final prosthesis was more than the resin stage. The difference between our study and the study by Kim et al is probably due to a lack of splint samples in our study.

In summary, according to our study, the axial displacement in hex group in the impression coping, resin abutment and final prosthesis was higher than other types when different torques were applied, with octagonal vertical displacement being the lowest, and morse hex 6° and morse hex 11° being between the two. Possible reasons are the difference in the angles used in this type of connection, and the difference in the level of the connection area where the force is exerted. Due to the importance of axial displacement in the miss fit, abutment-level impression is strongly recommended.

Because of the high cost of implant components, we used analogs instead of fixtures but further studies should be performed with fixtures to compare the results with the present results.

## Conclusions

In the present study, the following conclusions were drawn:

1. Vertical displacement at different stages of impression coping and resin abutment and final prosthesis had the highest and the lowest rates in hex and octagonal, respectively.
2. After the second tightening of 30 Ncm, repeated tightening showed almost constant settling values.

## Acknowledgments

This paper was written based on the thesis submitted by Dr. Gharekhani under the supervision of Dr.Saleh Saber (Thesis No.250̸ T).The authors would like to thankDrMehranMahbubkhah for providing the equipment for the tests.

## Authors’ contributions

FSS concepted the study and NA contributed to the study design. VG, SJA and MTM carried out the study procedures. VG drafted the manuscript. All the authorscritically revised the manuscript, read and approved the finalpaper.

## Funding

This study was supported and funded by the authors and dental and periodontal research center ofFaculty of Dentistry, Tabriz University of Medical Sciences

## Competing interests

The authors declare that they have no conflict of interest.

## Ethics approval

Not applicable.
